# Supplementary material for: Binaural summation of amplitude modulation involves weak interaural suppression
Source: Sci Rep. 2020 Feb 26;10:3560. doi: 10.1038/s41598-020-60602-5 (PMC7044261; doi:10.1038/s41598-020-60602-5)
Supplement: Supplementary file 1 — Supplementary Materials. [file 41598_2020_60602_MOESM1_ESM.docx]

Supplementary Materials

for

Binaural summation of amplitude modulation

involves weak interaural suppression

Baker, D.H.^1,2,6^, Vilidaite, G.^1,3^, McClarnon, E.^1^, Valkova, E.^1^, Bruno, A.^1^ & Millman, R.E.^4,5^

^1.^ Department of Psychology, University of York, Heslington, York, YO10 5DD, UK

^2.^ York Biomedical Research Institute, University of York, Heslington, York, YO10 5DD, UK

^3.^ School of Psychology, University of Southampton, University Road, Southampton, SO17 1BJ, UK

^4.^ Manchester Centre for Audiology and Deafness, University of Manchester, Manchester, M13 9PL, UK

^5.^ NIHR Manchester Biomedical Research Centre, Central Manchester University Hospitals NHS Foundation Trust, Manchester Academic Health Science Centre, Manchester, M13 9WL, UK.

^6.^ email: daniel.baker@york.ac.uk


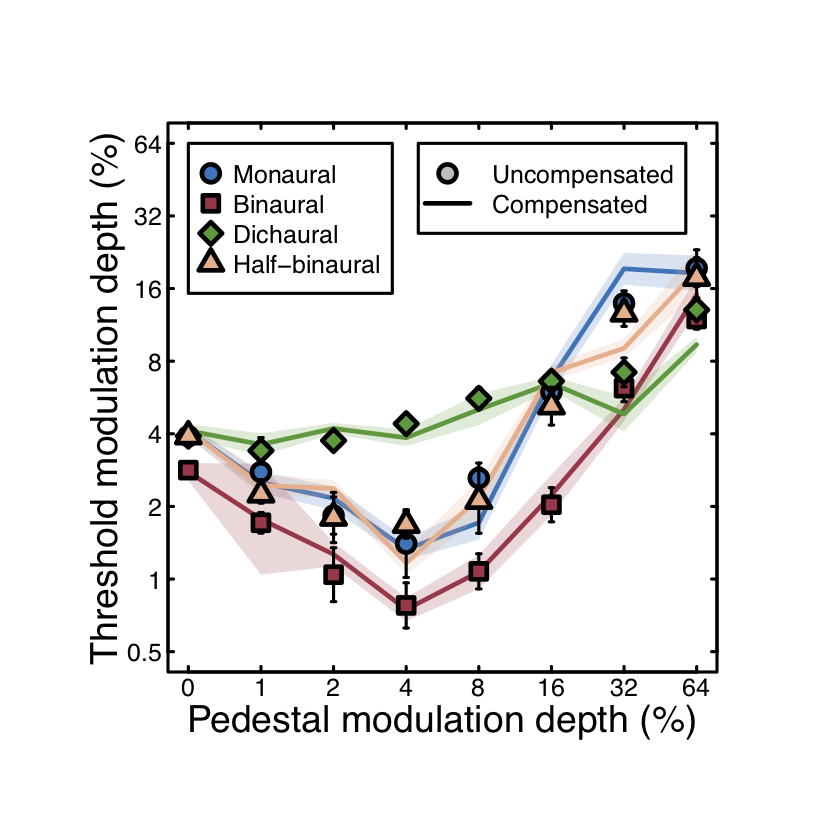


Figure S1: Comparison of thresholds for uncompensated (symbols) and compensated (lines) amplitude modulated stimuli, for one participant. We compensated for overall stimulus power using the method of Ewert and Dau^38^, in which each stimulus was scaled by $\sqrt{1+ m^{2}/2}$, where *m* is the modulation depth. Overall, the compensation had no systematic effect on thresholds. A paired t-test across all conditions produced no significant difference (*t*=0.26, *df*=31, *p*=0.79). Error bars and shaded regions give ±1SE of the Probit fit.


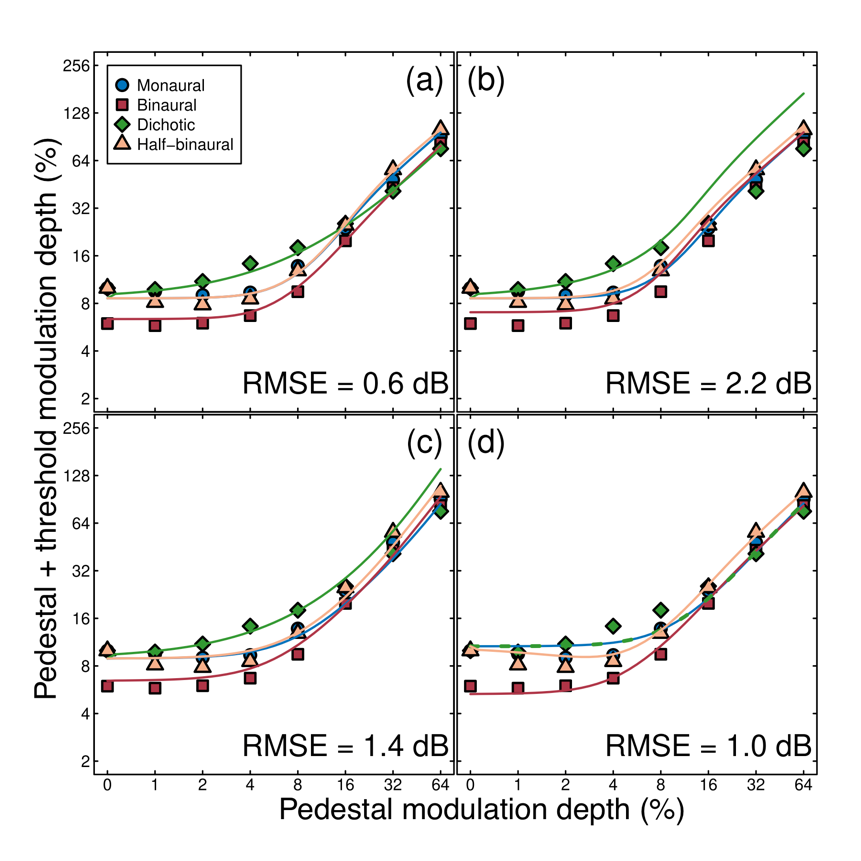


Figure S2: Alternative plotting of the data and model fits from Figure 6a-c (in panels a-c here) and 7a (in panel d here). In these plots, the y-axis represents the sum of the pedestal and threshold modulation depths. The model parameters are as given in Table 1. The RMS errors shown in each panel were calculated using the combined values of pedestal + threshold, converted into dB units (20*log_10_(100*(*m_pedestal_*+*m_threshold_*))). This plotting convention obscures the presence of the dip, and introduces spurious effects (e.g. the dichotic condition appears to show strong threshold elevation when in reality it is weak), but may be more familiar to some readers.
